# Oxoanion Imprinting Combining Cationic and Urea Binding Groups: A Potent Glyphosate Adsorber

**DOI:** 10.1021/acsomega.1c05079

**Published:** 2021-12-27

**Authors:** Sudhirkumar Shinde, Mona Mansour, Liliia Mavliutova, Anil Incel, Celina Wierzbicka, Hussein I. Abdel-Shafy, Börje Sellergren

**Affiliations:** † Biofilms Research Center for Biointerfaces, Department of Biomedical Sciences, Faculty of Health and Society, Malmö University, 20506 Malmö, Sweden; ‡ School of Consciousness, Dr. Vishwanath Karad MIT World Peace University, Kothrud, 411038 Pune, India; § Water Research & Pollution Control Department, National Research Centre, Dokki, 11727 Cairo, Egypt

## Abstract

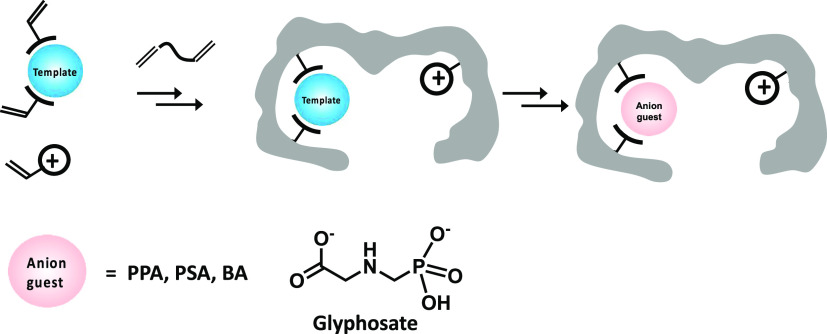

The use of polymerizable hosts in anion imprinting has led to powerful receptors with high oxyanion affinity and specificity in both aqueous and non-aqueous environments. As demonstrated in previous reports, a carefully tuned combination of orthogonally interacting binding groups, for example, positively charged and neutral hydrogen bonding monomers, allows receptors to be constructed for use in either organic or aqueous environments, in spite of the polymer being prepared in non-competitive solvent systems. We here report on a detailed experimental design of phenylphosphonic and benzoic acid-imprinted polymer libraries prepared using either urea- or thiourea-based host monomers in the presence or absence of cationic comonomers for charge-assisted anion recognition. A comparison of hydrophobic and hydrophilic crosslinking monomers allowed optimum conditions to be identified for oxyanion binding in non-aqueous, fully aqueous, or high-salt media. This showed that recognition improved with the water content for thiourea-based molecularly imprinted polymers (MIPs) based on hydrophobic EGDMA with an opposite behavior shown by the polymers prepared using the more hydrophilic crosslinker PETA. While the affinity of thiourea-based MIPs increased with the water content, the opposite was observed for the oxourea counterparts. Binding to the latter could however be enhanced by raising the pH or by the introduction of cationic amine- or Na^+^-complexing crown ether-based comonomers. Use of high-salt media as expected suppressed the amine-based charge assistance, whereas it enhanced the effect of the crown ether function. Use of the optimized receptors for removing the ubiquitous pesticide glyphosate from urine finally demonstrated their practical utility.

## Introduction

Molecular recognition, commonly associated with the precision exerted by biomacromolecule receptors when binding a ligand, can be challenged by the action of artificial receptors designed bottom-up by synthetic organic chemistry.^1,2^ Traditionally, host guest chemistry has focused on small-molecule binders comprising macrocyclic, cleft- or cage-like receptors featuring convergent binding groups complementary in size, shape, and electronic configuration to the incoming guest.^3−6^ Contrasting with these precisely defined receptors are molecularly imprinted polymers (MIPs) relying on the self-assembly principle.^7−12^ Functional monomers are allowed to interact with a template followed by polymerization in the presence of a crosslinking monomer. Subsequent removal of the template from the crosslinked polymer leaves behind recognitive sites with affinity for the template or a structural analogue. In the most common procedure, MIP affinity originates in a biomimetic way from multiple individually weak interactions between the functional monomer and the template. On the contrary, in host–guest-inspired imprinting, rationally designed host motifs are employed to target specific functional groups with higher affinity.^13−19^ This design principle has proven effective in the imprinting of small molecules or ions that can constitute substructures or epitopes of oligomeric or macromolecular targets.^19−21^ Prominent examples comprise MIPs targeting protein post-translational modifications, for example, phosphorylations, sulfations, and glycosylations.^19,22,23^ Inspired by organic crystal design and low-molecular weight hosts, we recently introduced polymerizable ureas acting as binary hydrogen bond donors with oxyanions such as phosphate, carboxylate, and sulfate.^24^ Hence, ternary complexes between a phosphoamino acid and a ureamonomer gave rise to MIPs capable of amino acid side chain-specific enrichments of phosphopeptides from endogenous samples. Recently, we reported these receptors to exhibit a unique sulfo/phospho-switching function of potential utility in phosphate/sulfate separations and scavenging.^25^ When combined with polymerizable crown ethers, the MIP urea receptors could be engineered to simultaneously recognize the oxyanion and its counterion.^26^ This concept was used to prepare phosphate receptors compatible with high-salt media. Inspired by these interesting results, we have probed here in more depth the parameters controlling receptor affinity and selectivity, notably the acidity and solubility of the urea monomer and the matrix polarity and means to stabilize or replace the counterion.

**Figure 1:**
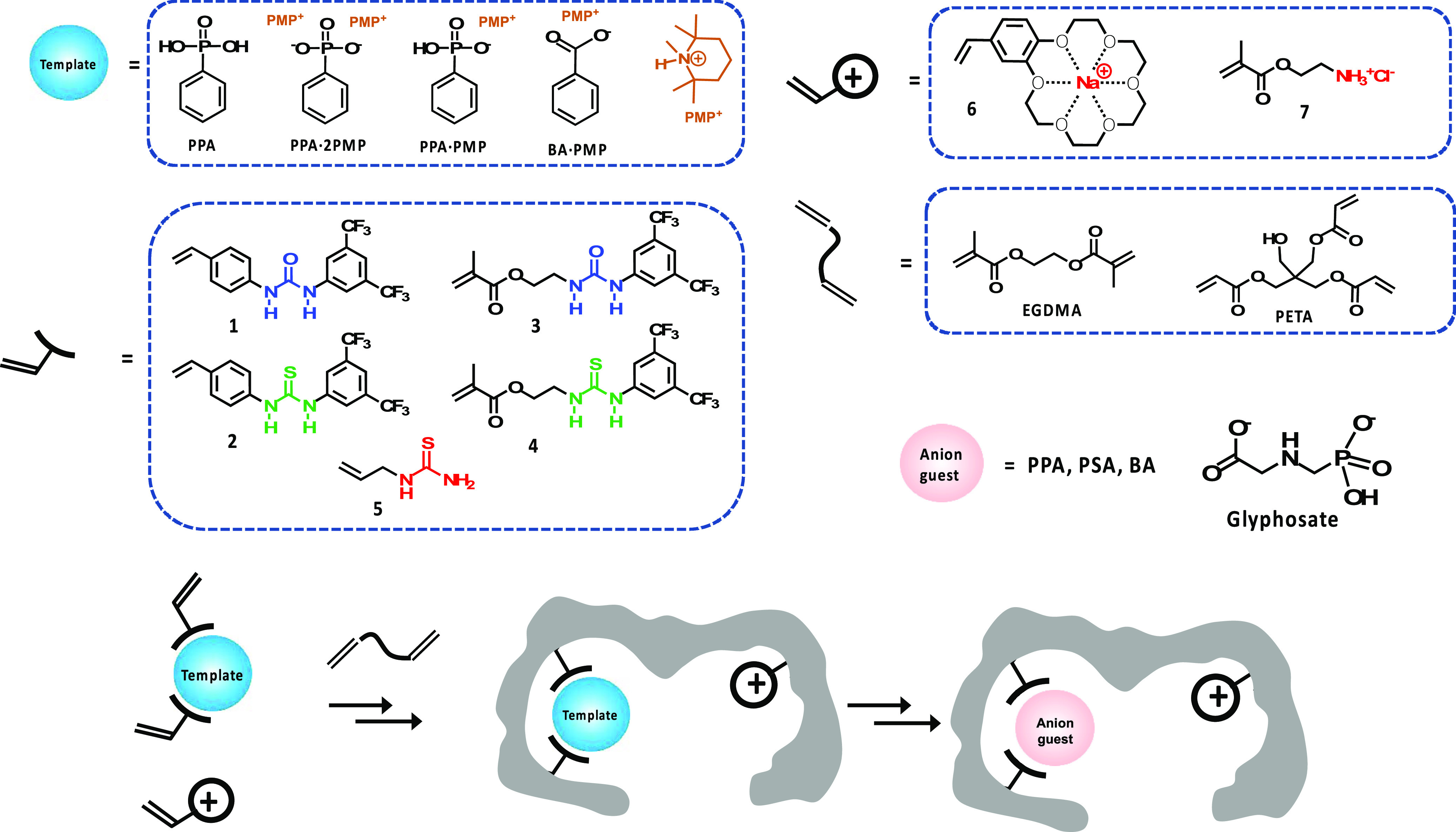
Templates, urea-based functional monomers (1–5), charged monomers (6 and 7), crosslinkers (EGDMA and PETA), and anion guests used to prepare and test the anion-imprinted polymer and scheme showing the preparation of a charge-assisted anion-imprinted binding site.

Using different templates and anion guests (Figure 1), the hydrogen bond donor capacity was probed by comparing oxoureas 1 and 3 with thiourea 2, 4, and 5, scaffold polarity was probed by comparing hydrophobic (EGDMA) and hydrophilic (PETA) crosslinkers, and additional charge stabilization was probed by introducing the polymerizable amine 7 or sodium binding crown ether 6. The insights gained will facilitate the rational design of anion hosts for a range of targets.

## Experimental Section

### Materials

Phenyl phosphonic acid (PPA) (98%), naphthylphosphoric acid (NP), and phenyl sulfonic acid (PSA) (90%) came from Aldrich (Milwaukee, USA), and benzoic acid (BA) (99%) came from Across-Organic. The acids were used after recrystallization from water. The base 1,2,2,6,6-pentamethylpiperidine (PMP) was purchased from Fluka (Buchs, Switzerland). Pentaerythritol triacrylate (PETA), ethylene glycol dimethacrylate (EGDMA), acrylamide (**8**), 2-aminoethylmethacrylamide (**7**), *N*-allylthiourea (**5**), and glyphosate were purchased from Sigma-Aldrich (Steinheim, Germany), whereas 18-crown-6 (18C6) and 4-vinylbenzo-18-crown-6 (**6**) came from Acros (Geel, Belgium). The initiator *N*,*N*′-azo-bis-(2,4-dimethyl)valeronitrile (ABDV) was purchased from Wako Chemicals (Neuss, Germany). Methanol (MeOH) of HPLC grade and MeCN of HPLC grade were purchased from Acros (Geel, Belgium). All anhydrous solvents were stored over appropriate molecular sieves. The water used in all experiments was Milli-Q water with a resistivity equal to 18.2 MΩ·cm. The mono-tetrabutylammonium (TBA) salt of NP was prepared as reported.^19^ The host functional monomer *N*-3,5-bis(trifluoromethyl)-phenyl-*N*′-4-vinylphenylurea (**1**) and *N*-3,5-bis(trifluoromethyl)-phenyl-*N*′-4-vinylphenylthiourea (**2**) were synthesized from 4-vinyl aniline (97%, Aldrich) and 3,5-bis (trifluoromethyl)-phenyl isocyanate (98%, Aldrich) and 3,5-bis (trifluoromethyl)-phenyl isothiocyanate (98%, Aldrich), respectively, as reported in the literature.^19^

### Apparatus

The HPLC measurements were carried out on an Agilent 1100 series HPLC instrument equipped with a reversed-phase column (Phenomenex Luna C-18, 250 × 4.6 (i.d.) mm) and photodiode array detector. FT-IR spectroscopy was performed using a NEXUS FT-IR spectrometer (Thermo Electron Corporation, Dreieich, Germany) equipped with an attenuated total reflection (ATR) accessory unit and ITR diamond (smart ITR) experimental setup. Scanning electron microscopy was conducted using an EVOLS 10 instrument from Zeiss in the high-vacuum mode and a secondary electron detector. The accelerating voltage was 15 kV, and the probe current was 50 pA. The working distance was 6.5–9 mm. The samples were glued to the sample stubs using Leit-C carbon cement and covered with gold using an Agar Scientific automatic sputter coater.

### 1-(4-Ethyl acrylate)-3-(3,5-bis(trifluoromethyl)phenyl)-urea (**3**)

To an ice-cooled solution of 2-aminoethyl methacrylate hydrochloride (0.663 g, 4 mmol) and triethylamine (0.558 mL, 4 mmol) in dry CH_2_Cl_2_ (30 mL), 3,5-bis (trifluoromethyl) phenyl isocyanate (0.692 mL, 4 mmol) was added slowly over 15 min under nitrogen followed by stirring of the reaction mixture at room temperature for 12 h. The mixture was washed with 1 M HCl (4 × 100 mL) and water (4 × 100 mL), and the organic phase was dried on anhydrous sodium sulfate. After drying, the organic layer was concentrated and purified by silica gel column chromatography using CH_2_Cl_2_/MeOH: 98/2 as an eluent. Evaporation gave 3 as a white solid (60% yield).

^1^H NMR (400 MHz, DMSO): δ 9.33 (s, 1H), 8.08 (s, 2H), 7.52 (s, 1H), 6.62 (t, 1H), 6.07 (s, 1H), 5.66 (s, 1H), 4.16 (t, 2H), 3.42 (q, 2H), 2.55–2.43 (m, 1H), 1.88 (s, 3H) (Figure S7).

### 1-(4-Ethyl acrylate)-3-(3,5-bis(trifluoromethyl)phenyl)-thiourea (**4**)

To an ice-cooled solution of solution of 2-aminoethyl methacrylate hydrochloride (0.663 g, 4 mmol) and triethylamine (0.558 mL, 4 mmol) in dry CH_2_Cl_2_ (30 mL) under nitrogen, 3,5-bis(trifluoromethyl) phenyl isothiocyanate (0.730 mL, 4 mmol) was added slowly over 15 min. Then, the reaction mixture was stirred at room temperature for 12 h. The mixture was washed with 1 M HCl (4 × 100 mL) and water (4 × 100 mL) followed by drying on anhydrous sodium sulfate. After drying, the organic layer was concentrated to give 4 as a white solid (65% yield).

^1^H NMR (400 MHz, CDCl_3_): δ 8.28 (s, 1H), 7.77 (m, 3H), 6.84 (s, 1H), 6.06 (s, 1H), 5.59 (s, 1H), 4.37 (t, 2H), 3.94 (s, 2H), 1.87 (s, 3H) (Figure S7).

### Preparation of Mono- and Disodium Salt of PPA (PPA·Na and PPA·2Na)

PPA·Na and PPA·2Na were prepared by equilibrating 1 and 2 equiv sodium hydroxide, respectively, in methanol with 1 equiv PPA followed by removal of the solvent and drying. The resulting white solid was dried in an oven at 40 °C for 12 h. The monosodium salt of PSA was prepared in a similar way.

### Studies of Complex Formation by ^1^H NMR Spectroscopy

^1^H NMR spectroscopic titrations were performed in dry deuterated DMSO. The association constant *K*_a_ for the interaction between hosts and guests was then determined by titrating an increasing amount of guest into a constant amount of functional urea monomer (*C*_0_ = 1 mM) with the amount of the added guest being 0, 0.25, 0.5, 0.75, 1.0, 1.5, 2.0, 4.0, 6.0, and 10.0 equiv with respect to the host. The complexation-induced shifts (CISs) of the host urea protons were followed, and titration curves were constructed with CISs versus guest concentration. The raw titration data were fitted to a 1:1 binding isotherm by non-linear regression using Prism 7 (Graphpad software Inc), from which association constants were calculated.

### Polymer Preparation

The functional monomers (color coded), templates, and crosslinkers shown in Figure 1 were used to create MIP/NIP libraries according to the design layed out in Table 1. Polymers were prepared at a 400 mg scale as exemplified for polymer P12 as follows.

**Table 1:**
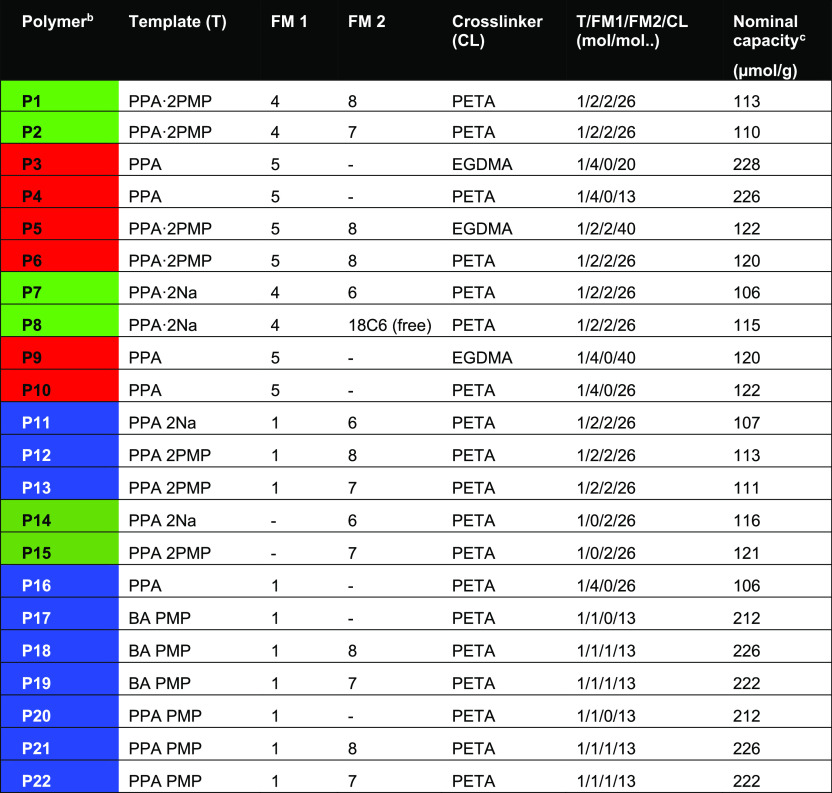
Combinatorial Polymer Library Composition^a^

a The polymers were prepared at a 400 mg scale as described in the Experimental Section using functional monomers (FM), crosslinkers, and templates as indicated (see Figure 1). Functional monomers: 1 = *N*-3,5-bis(trifluoromethyl)-phenyl-*N*′-4-vinylphenylurea; 2 = *N*-3,5-bis(trifluoromethyl)-phenyl-*N*′-4-vinylphenylthiourea acrylamide; 3 = 1-(4-ethyl acrylate)-3-(3,5-bis(trifluoromethyl)phenyl)-urea; 4 = 1-(4-ethyl acrylate)-3-(3,5-bis(trifluoromethyl)phenyl)-thiourea; 5 = *N*-allylthiourea; 6 = 4-vinylbenzo-18-crown-6; 7 = 2-aminoethylmethacrylamide; and 8 = acrylamide.

b The polymer numbers are color-coded referring to the type of FM1 urea host monomer used (1 = blue; 4 = light green; 5 = red; and none = dark green; see Figure 1).

c Defined as the amount in μmoles of added template divided by the total mass of polymer assuming quantitative monomer conversion.

PPA in the form of the bis-PMP (PPA·2PMP, prepared in situ) (0.05 mmol), **1** (0.1 mmol), AAm (0.1 mmol), and PETA (1.33 mmol) were dissolved in dry MeCN (0.61 mL). The initiator ABDV (4.4 mg, 1% w/w of total monomers) was added to the solution, which was subsequently transferred to a screw cap vial, cooled to 0 °C, and purged with a flow of dry nitrogen for 10 min. The vials were sealed with silicon insulating tape and kept in an oven at 50 °C for 24 h and subsequently at 80 °C for 3 h. The vials were thereafter broken, and the polymers were crushed in a mortar with pestle for further use. Non-imprinted polymers (NIPs) were prepared in the same way as described above but with the omission of the template molecule from the pre-polymerization solution.

To promote template removal, the polymers were shaken overnight in MeOH/1 N HCl (80:20), followed by centrifugation and HPLC analysis of the supernatant for the released template. The extraction was repeated twice for 3 and 1 h while monitoring the extent of template removal. Thereafter, the polymers were washed in Milli-Q water and MeOH. All wash fractions were analyzed by HPLC for the released template.

### Reversed-Phase HPLC Detection of Unbound PPA and PSA

Chromatographic analysis was carried out with an HPLC 1100 instrument (Agilent) equipped with a quaternary pump auto-injector and DAD detector. The analytical column was a polar end capped C18 reversed-phase column (Phenomenex Luna 250 mm × 4.6 mm). The mobile phase consisted of methanol/water (0.1% TFA): 32/68 (v/v), and the flow rate was 1 mL/min. The injection volume was 5 μL. The column was kept at room temperature. The absorbance wavelength was 205 nm for PSA and 225 nm for PPA. For quantification purposes, calibration standards were made up using the same solvent as used in the binding experiments. The calibration curve comprised the 0–1.2 mM concentration range.

### Testing of Polymer Uptake of Different Anions

The imprinted and nonimprinted polymers (10 mg) were incubated by shaking in solutions of the different anions for 15 h. The solutions were centrifuged, and the supernatant was analyzed by HPLC for detecting unbound anions. The amount of bound anions (*B* μmol/g) was determined as
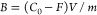
1where *C*_0_ (mM) is the initial concentration of anions, *F* (mM) is the free concentration in the supernatant, *V* (mL) is the volume of the sample, and m is the weight of the polymer (g). The imprinting factors IF were calculated as given in eq 2
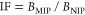
2where *B*_MIP_ and *B*_NIP_ are the amount of bound anions by the MIP and NIP, respectively.

### Binding Isotherms

The polymers (10 mg each) were incubated in 1 mL solutions of the anions at different concentrations. The vials were shaken for 24 h followed by centrifugation and quantification of the unbound analyte by HPLC as described above. The amount of the bound analyte per unit mass of imprinted polymer (*B*_MIP_) was calculated according to eq 1 and corrected for binding to the non-imprinted polymer as *B* = *B*_MIP_ −*B*_NIP_. Each experiment was performed at least twice. Binding curves were constructed by plotting *B* against free concentration *c* and were subsequently fitted by non-linear regression using GraphPad Prism 7 software (GraphPad Software, La Jolla, CA, USA) to a Hill binding site model (eq 3)
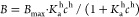
3where *c* is the free concentration of solute, *h* is the Hill slope, *B*_max_ is the corrected maximum amount of solute bound by the polymer particles at saturation, and *K*_a_ is the association constant.

### Solid-Phase Extraction Experiments

Polymers were ground to fine particles, and 40 mg of each one was packed in single-frit (20 μm) cartridges. Solutions (1 mL) of PPA, PSA, or glyphosate (1 mM) were allowed to percolate through the columns whereafter the free anion concentrations in the receiving solution were measured using HPLC. Regeneration of the columns was performed by washing with MeOH/1 N HCl (80:20) followed by water.

### SPE of Glyphosate in Urine

A urine diversion toilet was implemented in the National Research Centre pilot plant in Cairo, Egypt. Urine was directed through a piping system to a collection tank. The main characterized parameters of urine were measured according to the Standard Methods for Examination of Water and Wastewater (APHA) (American Public Health Association, 2005). Urine samples were allowed to percolate through the cartridges as described above but using a sorbent weight of 100 mg.

### Reversed-Phase HPLC Detection of Unbound Glyphosate

The analytical column was an Eclipse XDB-C18 RPLC column (50 × 4.6 mm i.d.). The HPLC analysis was conducted by gradient elution using 0.1% formic acid in water as mobile phase A and acetonitrile as mobile phase B. The flow rate was 1 mL/min, and the injection volume was 200 μL. The column was kept at room temperature. The absorbance wavelength was 208 nm. For quantification purposes, calibration standards were made up using the same solvent as used in the binding experiments.

## Results and Discussion

### Host Monomer Design and Counterion Effects

The ample use of the urea and thiourea motifs in anion receptor design reflects their finely tunable hydrogen bond donor capacity combined with a straightforward synthesis.^6,27,28^ With respect to oxoanions, the parallel arrangement of the two NH donors leads to stable eight-membered ring structures held together by two linear hydrogen bonds. In the absence of proton transfer, the affinity for a given oxoanion increases with the acidity of the urea protons, which can be finely adjusted by introducing alkyl or aryl substituents with appropriate electron-withdrawing groups (EWGs). Hence, introducing alkyl substituents such as in urea monomers 3 and 4 (Figure 1) reduces affinity, whereas aryl substituents featuring EWGs such as in 1 and 2 (Figure 1) typically lead to enhanced affinity. In addition, the donor–acceptor complex stability can be tuned by replacing the oxourea with a thiourea. The thiourea hydrogens are more acidic and more susceptible to deprotonation but typically interact more strongly with less basic oxoanions.^29^ Thioureas moreover are better soluble than oxoureas (reflecting weaker self-association) and are preferred in aqueous solvent systems. Since the oxoanion carries a countercation, the latter presents yet a tunable parameter when optimizing urea-based anion receptors. As we have shown in previous reports, lipophilic cations in the form of aprotic quaternary ammonium ions^23^ or Na·18C6^26^ prevent contact ion pairing, which in turn leads to stronger complexation with the urea donor and enhanced imprinting. As also proven in bottom-up host design, combining precisely placed charges with the urea donor in controlled microenvironments, results in oxoanion hosts compatible with aqueous solvent systems. This latter design principle fits perfectly with molecular imprinting due to the self-assembly-driven placement of the interacting binding groups. In order to verify the abovementioned trends in terms of urea donor capacity, we investigated monomers 1–4 with respect to their complex stability with the mono TBA salt of naphthylphosphoric acid (NP·TBA). The interaction strengths assuming a 1:1 interaction model was investigated by ^1^H NMR titrations in DMSO-*d*_6_. Complex stability constants were calculated using the induced downfield shifts of the urea protons (Figure S1) and are given in Table 2. With respect to monoanions, oxourea 1 formed the more stable complex followed by thiourea 2, thiourea 4, and oxourea 3. This trend can be explained based on the abovementioned arguments and conformation effects. The 1,3-diaryl monomers (1 and 2) are more acidic and hence interact more strongly with the guest compared to alkyl–aryl counterparts (3 and 4). Furthermore, thiourea monomer 4 interacted more strongly with the guest compared to urea analogue 3 as expected based on the difference in acidity of ureas and thioureas (*vide supra*). However, this relationship did not hold true for 1 and 2 pairs of monomers. This can possibly be ascribed to a preference of diaryl thioureas to adopt a (Z,E) conformation as opposed to preferential (Z,Z) conformation of diaryl ureas.^30^ Based on these results, we decided to compare the monomers in oxoanion imprinting.

**Table 2:** Association Constants for Complexes of Naphthylphosphate Monoanion (TBA Salt) and Functional Monomers in DMSO-*d*_6_^a^

functional monomer	*K*_a_ (M–1)	CIS (ppm)
1	1489 ± 7	2.14
2	743 ± 10	1.96
3	266 ± 14	1.91
4	439 ± 5	2.00

a The parameters were determined by ^1^H NMR titrations from the average of the individual CISs of both urea protons.

### Polymer Preparation and Template Rebinding Tests

To study the influence of the above-listed parameters in the design of oxoanion receptors, we included oxourea and thiourea monomers 1 and 4 in combination with charged (6, 7) and neutral (8) comonomers, and different crosslinkers and thereafter assessed template rebinding to the resulting polymers in non-aqueous and aqueous solvent systems. Thiourea 5 was included since it has been used as a functional monomer in phosphate binding MIPs^31^ and in commercial solid-phase extraction (SPE) materials targeting glyphosate.^32^ Two crosslinkers were compared, difunctional EGDMA and trifunctional PETA, the latter featuring a pendent hydrophilic methylol group. The initial monomer feed ratios were chosen based on previous reports.^25,26^ Imprinted and non-imprinted polymers were prepared at a 400 mg scale according to the monomer/template compositions given in Table 1, ground to course particles, and freed from the template by exhaustive solvent extraction. To evaluate the template rebinding properties, the particles of polymers P1–P16 were incubated with solutions of PPA in the form of free acid or its disodium salt. The influence of solvents and additives was studied by including binary mixtures of acetonitrile and water with or without TFA and 1 M NaCl in pure water. Specific binding of the solutes was subsequently calculated based on HPLC quantifications of unbound fractions, as described in the Experimental Section.

**Figure 2:**
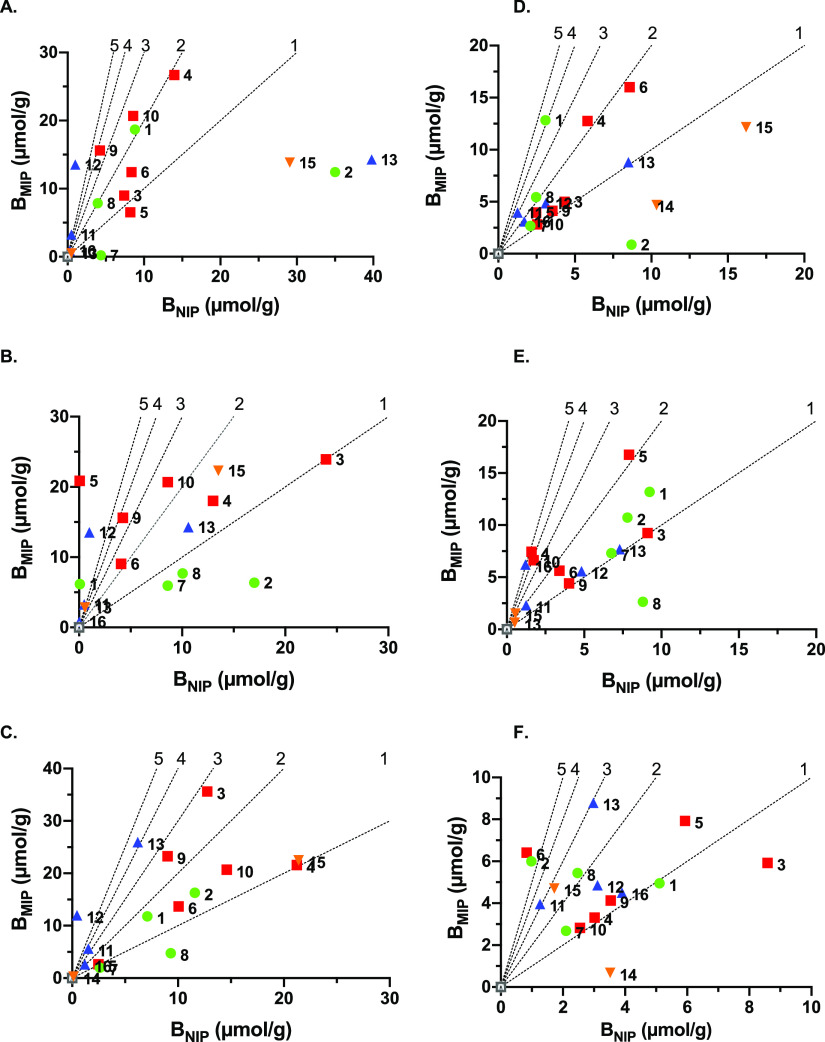
Amount of PPA (free acid) bound to polymers from the library prepared according to Table 1 after incubation of the polymers in a solution of PPA (0.6 mM) in MeCN (A,D); MeCN/water: 50/50 (v/v) (B,E); or water (C,F) in the absence (A–C) or presence (D–F) of 0.1% TFA. The diagonal lines represent the theoretical IF values of 1, 2, 3, 4, and 5. The MIP/NIP couples are represented by numbers 1–16 corresponding to the polymer numbering and functional monomer color codes of Table 1.

**Figure 3:**
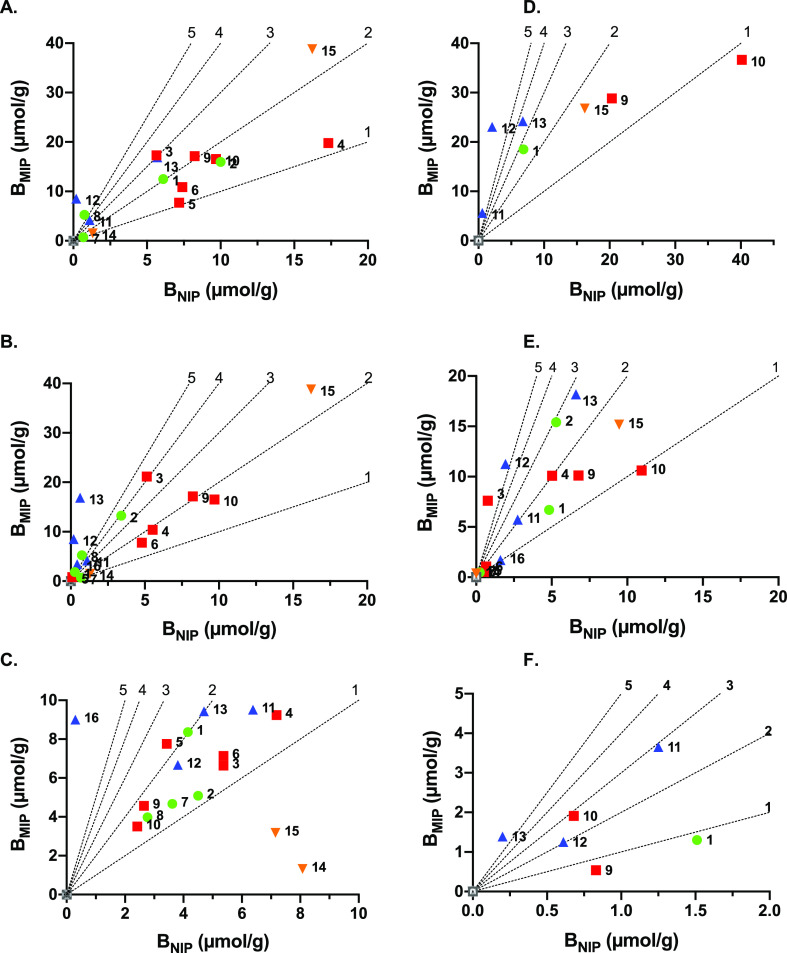
Amount of PPA·2Na (A–C) and PSA·Na (D–F) bound to polymers from the library prepared according to Table 1 after incubation of the polymers in a solution of the analytes (0.6 mM) in MeCN/water: 50/50 (v/v) (A), water (B,D), water (0.1% TFA) (E), and 1 M NaCl (C,F). The diagonal lines represent the theoretical IF values of 1, 2, 3, 4, and 5. The MIP/NIP couples are represented by numbers 1–16 corresponding to the polymer numbering and functional monomer color codes of Table 1.

Figures 2 and 3 show graphs displaying MIP versus NIP binding corresponding to the polymer compositions in Table 1 with color coding matching the structure of the urea group in the polymer. As a first general observation, binding to MIPs exceeds that of the NIP for most polymer compositions. This is reflected in the imprinting factors (Ifs), easiest estimated from the slope of the nearest line crossing origo.

#### Comparing crosslinkers

First, the graph representing binding of PPA free acid in acetonitrile is considered (Figure 2A,D). Highest PPA uptake is shown here by the monomer 5 MIPs (red squares) with polymers prepared using the polar crosslinker PETA (P4, P6, and P10) showing an uptake exceeding that of their EGDMA counterparts (P3, P5, and P9). This behavior is reversed when increasing the water content to 50% (Figure 3B,E) with the effect being even more pronounced in pure water (Figure 2C,F), notably for P3 and P5 versus P4 and P6. We tentatively ascribe this result to a transition from electrostatically to hydrophobically driven binding.^33^ Lacking strong solvation, solute binding is driven by direct electrostatic interactions with the polymer binding sites somewhat akin to the chromatographic “normal-phase” retention mode with binding reinforced by the use of polar crosslinkers such as PETA (cf. silanol groups). Binding in water on the other hand is in part hydrophobically driven given the apolar phenyl group of the solute. This contribution is magnified by the use of less-polar polymer scaffolds (cf. EGDMA). Also, of note concerning the monomer 5 MIPs is that the uptake scales with template load, that is, P3 and P4 feature 2× higher nominal site density than P5 and P6, respectively, and P9 and P10, respectively.

#### Comparing Urea Monomers

We then turned to comparing the two thioureas 4 (green circles) and 5 (red squares) and oxourea monomer 1 (blue triangles). P1 (monomer 4), P6 (monomer 5), and P12 (monomer 1) are directly comparable, having identical compositions with the exception of the urea monomer. Considering the two thiourea monomers, in nearly all solvent systems, P1 features both a higher specific binding and imprinting factor compared to P6. Apart from the affinity per se for the oxyanion, the polymer primary structure is affected due to the different reactivities of the two monomers. In contrast to methacrylic monomer 4, allylic monomers such as 5 are poorly reactive and only add to the chains after the more reactive acrylic or methacrylic monomers are consumed.^34^ In most solvent systems, P12 prepared using the oxourea monomer 1 binds comparable or slightly larger amounts of solute than P6 (see Figures 2A–C,E and 3A–C). More striking is the higher imprinting factors of P12, which exceed those of 5 in Figures 2A–C and 3A,B,D,E. Given equal uptakes noted for the MIPs, this is due to lower NIP binding, possibly as a result of masked urea functionalities.

#### Influence of Charged Comonomers

Two monomers (6 and 7) were compared to test whether introduction of positively charged groups in vicinity of the host monomer would enhance binding affinity. Both of the monomers were anticipated to form ion pairs with the template (PPA·2Na) prior to polymerization, amine 7 by ion exchanging with Na^+^ and crown ether 6 by complexing Na^+^. Neutral polymers P1 and P12, prepared using acrylamide (8) as a comonomer, should be compared with 7 containing P2 and P13 and 18C6 containing P7 and P11, respectively. Moreover, P14 and P15 prepared identically as P11 and P13, respectively, but in the absence of urea monomer 1 were included to gauge the effect of the comonomer alone. As seen in both Figure 2 (PPA free acid) and Figure 3 (PPA and PSA sodium salt), the effect of amine 7 was strongly solvent-dependent.

In pure acetonitrile (Figure 2A), binding to the non-imprinted polymers P2 and P13 and control polymer P15 strongly exceeds binding to the corresponding MIPs, and the effect persists to some extent in the presence of the acid modifier TFA (Figure 2D). Interestingly, this behavior is completely reversed in water, Figures 2C,F and 3A–C, where instead, the MIPs feature enhanced PPA uptake. We tentatively ascribe this effect to different polymer structures and microenvironments of the charged groups. Reactivity ratios of charged monomers strongly depend on counterions and solvent, suggesting that these MIPs and NIPs feature different primary structures. Moreover, assuming the template to coordinate both the amine and the urea group in a hydrophobic binding pocket (vide supra), binding of PPA to this pocket is favored by raising the aqueous content. This contrasts with the NIP lacking this prearrangement, presumably leading to a more random and solvent-accessible functional group arrangement. The latter is more susceptible to water and competing ions.

Figures 2C,F and 3C show the effect of added salt on the charge-assisted binding. While in the absence of salt, PPA binding to the amine 7 containing polymers P2 and P13 exceeds the binding to P1 and P12 lacking 7, the effect vanishes in the presence of 1 M NaCl. Moreover, the lower uptake to P15 in relation to P13 (cf. Figure 2C,F) supports the cooperativity between the two functional monomers. In the presence of salt (Figure 3C,F), the effect of the charged group is strongly diminished. A completely different trend is observed when introducing crown ether monomer 6 as a charge-carrying comonomer. Compared to all polymers, polymer P11 displayed weak binding of PPA in all solvent systems except for in the high-salt solution (1 M NaCl) (Figure 3C), where it showed the highest uptake.

This effect is also manifested for PSA binding, as shown in Figure 3F. The MIP containing the crown ether monomer alone (P14) shows weak binding in all solvent systems, again proving the cooperative action of the two functional monomers. As discussed in our previous report,^26^ sodium complexation by 18C6 in water requires elevated sodium concentrations and is hence absent in the other solvent systems. Lacking charge assistance, these polymers (P7 and P11) display only a low affinity for the oxoanions. For a more in-depth characterization, we scaled up thiourea polymers P1, P9, and P10 and compared them with urea polymers P11 and P12.

#### Monovalent Anions as Templates

**Figure 4:**
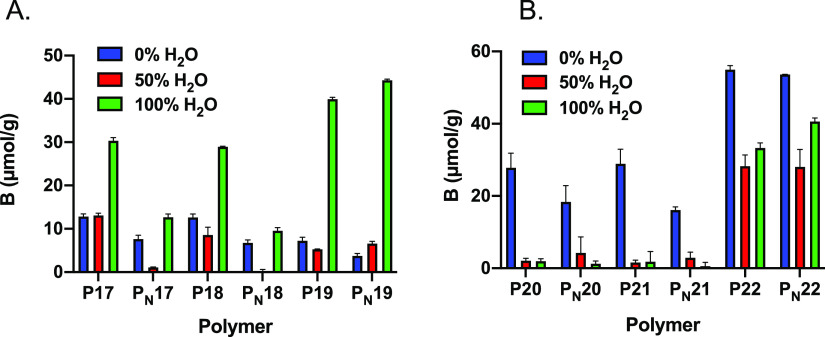
Specific binding of BA·PMP (A) and PPA·PMP (B) to imprinted and non-imprinted polymers according to Table 1 in MeCN water mixtures.

To investigate how charged comonomers affected binding of monovalent anions, six more polymers were prepared using BA·PMP and PPA·PMP as monovalent templates (P17–P22). Polymers were prepared in the absence of the comonomer, in the presence of acrylamide 8, and in the presence of amine monomer 7. Considering first the tests of the PMP·PPA-imprinted polymers in acetonitrile (Figure 4B), the imprinting factors for P20 and P21 are lower than those for P12 prepared using the divalent template (cf. Figure 2A). Interestingly, binding is nearly completely suppressed in the presence of water, most likely as a result of the weaker complexation of the PPA monoanion compared to the PPA dianion.^25^ Introduction of the charged group as in P22 overall enhances binding but completely suppresses the imprinting effect. This is seen in all solvent systems and is different from the behavior of the PPA·2PMP-imprinted P13, which displays significant imprinting in all aqueous solvent systems (cf. Figures 2 B,C,E,F and 3). In the case of BA (Figure 4A), the results are somewhat different. The BA anion is a stronger base than the PPA monoanion and complexes 1 more strongly (*K*_eq_ = 8820 M^–1^ vs *K*_eq_ = 7005 M^–1^, respectively, in deuterated DMSO). This disagrees with the weaker binding shown by the BA-imprinted polymers P17–P19 compared to the PPA counterparts. In MeCN, the specific binding to the former is less than half of that to the latter. Increasing the aqueous content however reverses the picture with the BA polymers displaying distinct template binding and imprinting. This behavior we tentatively explain by electrostatic and solvation effects. In MeCN, in spite of carboxylate former stronger complexes with 1, the phosphonate exhibits a larger molecular volume and one additional OH group capable of interacting electrostatically with the polymer scaffold. In the presence of water, the relative hydration energies of the two monoanions seem instead to be the dominating factor. Assuming monoanions of inorganic carbonate^35^ and phosphate^36^ as estimates (HCO_3_ = −380 kJ/mol; H_2_PO_4_ = −522 kJ/mol), BA is less strongly hydrated than PPA, for which the strong hydration will prevent its interaction with the binding site.

### Adsorption Isotherms and Binding Parameters of Selected Polymers

**Figure 5:**
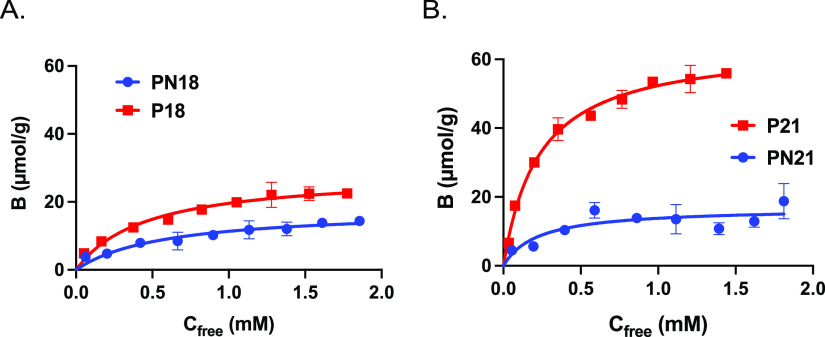
Equilibrium binding isotherms for (A) BA·PMP binding to polymer P18/PN18 and of (B) PPA·PMP to polymer P21/PN21 in MeCN.

The binding energy distributions of the polymers are given by single-component adsorption isotherms determined by batch equilibration in acetonitrile (Figures 5 and S2), 1 M NaCl (Figure S3), and 0.1 M sodium bicarbonate buffer at pH 9 containing 1 M NaCl (FigureS 4). To confirm the relative binding affinity of the two monoanions of BA and PPA, we first measured the binding isotherms for their mono PMP salts on P18 and P21 (Figure 5). The isotherms were best fitted with the Langmuir monosite binding model, resulting in the binding parameters shown in Table S1. In accordance with the discussion given above, BA forms weaker interactions with its polymer complement with a significantly lower saturation capacity compared to PPA.

**Figure 6:**
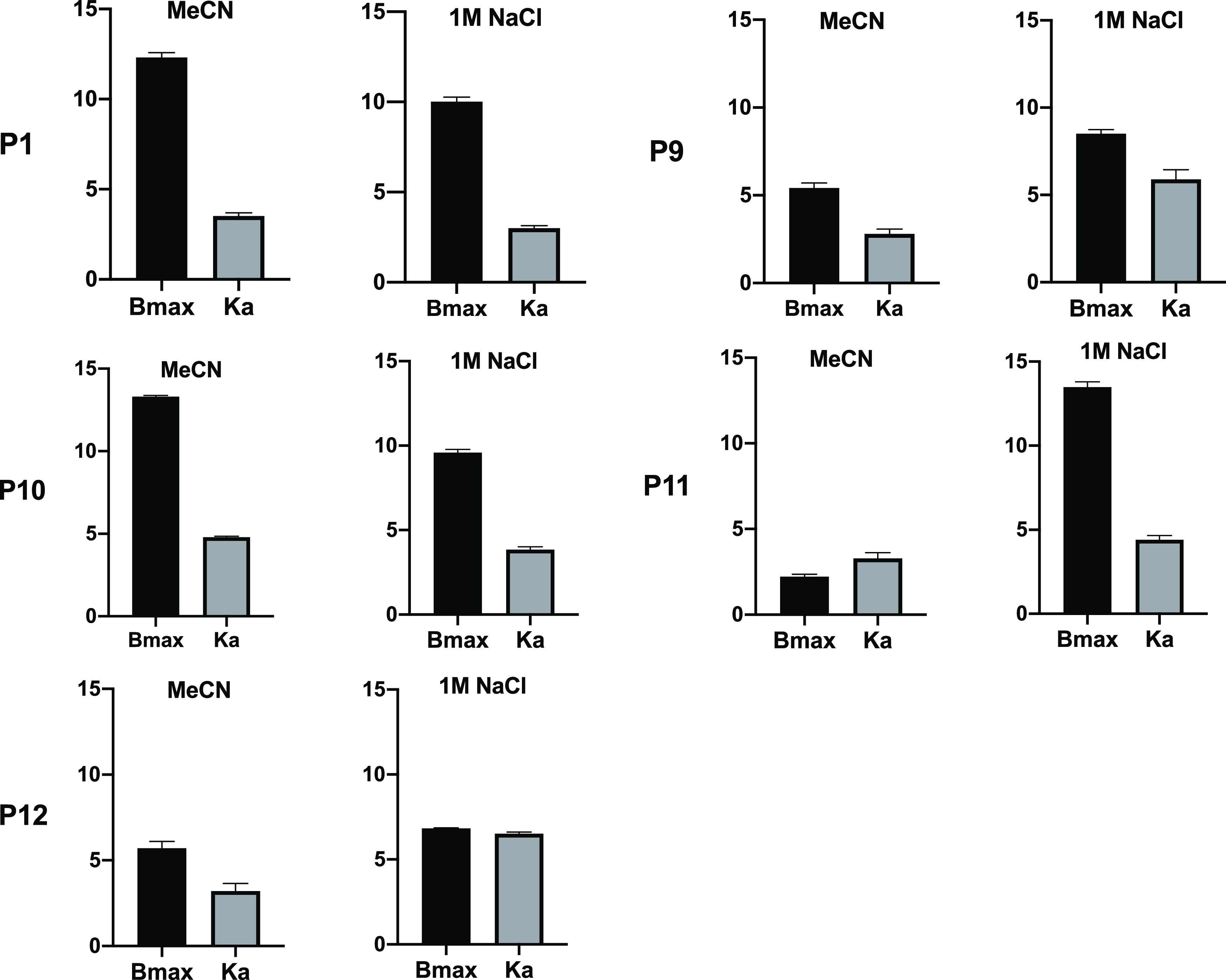
Association constant *K*_a_ (×10^3^ M^–1^) and saturation capacity *B*_max_ (μmol/g) for the binding of PPA to the imprinted polymers defined in Table 1 in MeCN or 1M NaCl. PPA was added in the form of free acid (MeCN) or its disodium salt (1 M NaCl). Binding data corrected for binding to the corresponding NIPs.

This contrasts with the latter featuring a high saturation capacity, in agreement with our previous report.^25^ The isotherms of P1 and P10 featured a pronounced sigmoidal shape and were therefore fitted to the Hill equation describing a cooperative binding model. This resulted in the binding constants (*K*_a_) and saturation capacities (*B*_max_) given in Figure 6 and Tables S2 and S3.

Considering first the acetonitrile results (Figure S2), we gratefully noted that both *K*_a_ and *B*_max_ increased in the same order as the uptakes in Figure 2A with P11 < P12 ≈ P9 < P1 ≈ P10, again confirming the “normal” versus “reversed-phase” behavior of P9 and P10. Considering instead the binding curves obtained when incubating in 1M NaCl, the behavior was different. With the exception of P12, both *B*_max_ and *K*_a_ of the PETA polymers P1 and P10 dropped with respect to the MeCN results. This contrasted with the EGDMA polymer P9, which displayed a significant increase in both *K*_a_ and *B*_max_. The most pronounced effect of the high-salt incubation was the strongly enhanced binding (ca 5× increase) to P11. Binding to P12, identically composed as P11 but lacking the crown ether moiety, was meanwhile not affected by the solvent switch. This confirms that our dual-ion receptor approach is effective in boosting anion binding affinity in high-salt media.

**Figure 7:**
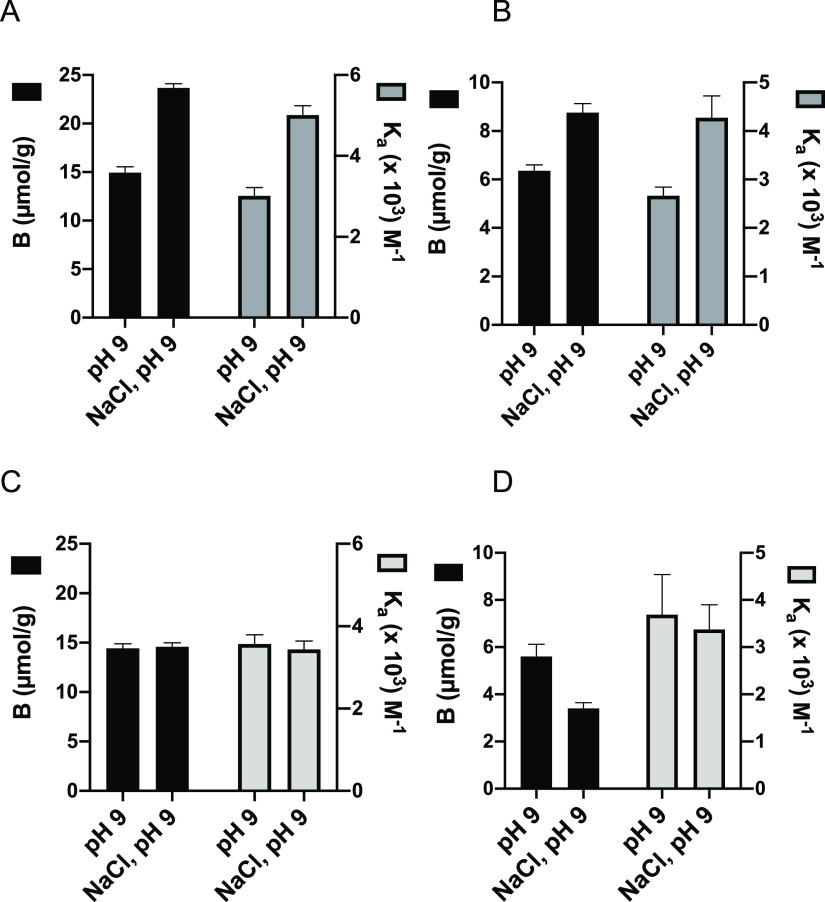
Association constant *K*_a_ and saturation capacity *B*_max_ for the binding of PPA·2Na (A,C) and PSA·Na (B,D) to imprinted polymers P11 (A,B) and P12 (C,D) according to Table 1 in pH 9 sodium bicarbonate buffer in the absence and presence of 1 M NaCl. Binding data corrected for binding to the corresponding NIPs.

According to our previous report, binding of PPA, PSA, and inorganic salts is enhanced at alkaline pH where the acids are fully ionized. Hence, we determined the binding isotherms for PPA and PSA binding to P11 and P12 in a pH 9 buffer, both in the absence and presence of 1 M NaCl (Figure 7). The results essentially confirm the abovementioned conclusions. The effect of the pH adjustment is a nearly twofold-increased *B*_max_. Addition of salt leads to an increase in *B*_max_ and *K*_a_ for both PPA and PSA on P11, whereas no or a suppressive effect is observed for P12.

As expected from the initial batch binding experiments in Figure 2A, the saturation capacity for PPA is overall higher than that for PSA. Fitting these data with the one-site host–guest model resulted in the curves shown in Figure S6 with the fitting parameters *K*_a_ and *B*_max_ given in Table S3. The preference for PPA is reflected in the higher association constants recorded for this anion again with the highest values obtained for the imprinted polymers.

### Polymer Physical Characterization

To confirm identity and chemical compositions, upscaled polymer batches of P9, P11, P12, and P13 were subjected to physical characterization. The SEM images in Figure S5 revealed rough textures that were similar for all polymers in support of a mesoporous morphology. Meanwhile, the transmission FTIR spectra (Figure S6) showed all characteristic bands with no apparent difference between imprinted and non-imprinted polymers, all in all indicating a stoichiometric monomer incorporation and successful template removal from the imprinted polymers.

### Use of Oxoanion MIPs for Glyphosate Removal

**Figure 8:**
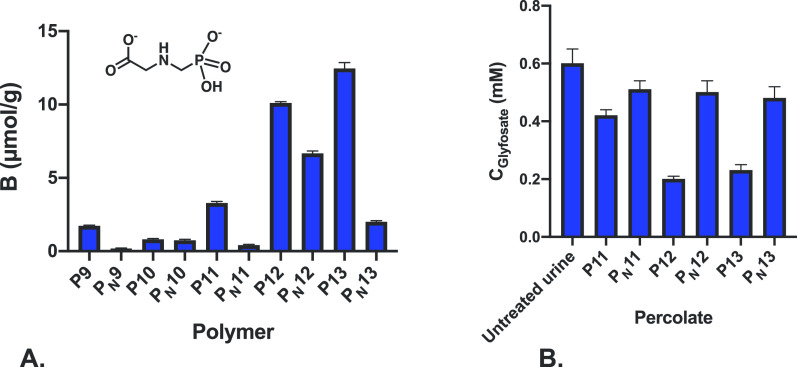
(A) Specific binding of glyphosate to imprinted and non-imprinted polymers according to Table 1 in water and (B) concentration of glyphosate in a urine sample spiked with 0.6 mM glyphosate after percolation through SPE columns packed with PPA-imprinted and non-imprinted polymers.

In order to finally demonstrate a practical application of the phosphate binding polymers, we turned to the ubiquitous pesticide glyphosate. Glyphosate is the world’s most heavily used pesticide ever. Its frequent use in agriculture is due to its effectiveness in controlling weeds that can otherwise persist for years.^37^ Approximately 9.4 million tons has been sprayed on crops worldwide since its introduction 1974 and the usage continues to rise largely due to the use of pesticide/herbicide-resistant GMO crops. Although glyphosate is claimed to be largely inert and excreted from the body, numerous studies report high levels of the pesticide in urine samples, blood samples, and breast milk. As for polar pesticides, glyphosate is very challenging both with respect to removal and quantification, and this calls for new solutions.^38^ To test whether the optimized urea-based phosphate receptors would serve this purpose, we first included thiourea-based polymers P9 and P10, both prepared using the thiourea monomer used in the commercially available MIP sorbents.^32^ These polymers were compared with the oxourea MIPs P11, P12, and P13. As seen in Figure 8A, the uptake of glyphosate from an aqueous solution varied significantly between the polymers. While the thiourea MIPs P9 and P10 showed uptakes of less than 3 μmol/g, the oxourea MIPs P12 and P13 showed pronounced imprinting and specific binding of more than 10 μmol/g. The best binders P11–P13 were therefore taken to a realistic removal test using artificially contaminated urine. A urine sample was first collected from a urine diversion toilet (see the Supporting Information) and spiked with glyphosate at 0.6 mM (Table S4). A noticeable increase in the COD was recorded, accompanied by a slight decrease in pH. Subsequently, the aliquots of the sample were allowed to percolate through the MIP-SPE columns under gravity followed by characterization of the percolates. Table S4 and Figure 8B show a pronounced influence of imprinting on the level of glyphosate scavenged. In agreement with the results in Figure 8A, P11 performed relatively poorly, whereas SPE on P12 and P13 produced a nearly threefold lowering of the glyphosate concentration.

## Conclusions

Based on a detailed experimental design of phenylphosphonic and benzoic acid-imprinted polymer libraries using urea- or thiourea-based host monomers in the presence or absence of cationic comonomers, we demonstrated here powerful receptors capable of oxyanion recognition in non-aqueous, fully aqueous, or high-salt media. While the affinity of thiourea-based MIPs increased with the water content, the opposite was observed for the oxourea counterparts. Binding to the latter could however be enhanced by raising pH or by the introduction of cationic amine- or Na^+^-complexing crown ether-based comonomers. Use of high-salt media as expected suppressed the amine-based charge assistance, whereas it enhanced the effect of the crown ether function. The work shows the importance of fully exploring the compositional parameters of MIPs in order to arrive to an enhanced target affinity and selectivity. To demonstrate the payoff for these efforts, we addressed a real-life environmental pesticide pollution problem. Outperforming commercial benchmarks, our best binders were capable of effective reduction of the level of glyphosate from urine, demonstrating the practical utility of these receptors. We believe that the insights gained in this work will facilitate the rational design of anion hosts for a range of targets.

## Supporting Information Available

Experimental section outlining synthetic procedures, characterization techniques, and data evaluation and dynamic simulations (PDF)
