# Supplementary material for: Oxoanion Imprinting Combining Cationic and Urea Binding Groups: A Potent Glyphosate Adsorber
Source: ACS Omega. 2021 Dec 27;7(1):587–98. doi: 10.1021/acsomega.1c05079 (PMC8757333; doi:10.1021/acsomega.1c05079)
Supplement: Supplementary file 1 — ao1c05079_si_001.pdf [file ao1c05079_si_001.pdf]

Supporting Information

Oxoanion imprinting combining cationic and urea binding groups: A potent glyphosate adsorber

Sudhirkumar Shinde,[#1,2] Mona Mansour,[#1,3] Liliia Mavliutova,[1] Anil Incel,[1] Celina Wierzbicka,[1] Hussein I. Abdel Shafy[4] and Börje Sellergren*[1]

1) Department of Biomedical Sciences, Faculty of Health and Society, Malmö University 20506 Malmö, Sweden.
2) School of Consciousness, Dr Vishwanath Karad Maharashtra Institute of Technology – World Peace University, Kothrud, Pune 411038, India
3) Current address: Analysis and evaluation department,
   Egyptian petroleum research institute, 1 ahmed el zomor street, nasr city,
   Cairo, Egypt
4) Water Research & Pollution Control Department, National Research Centre, Dokki, Cairo, Egypt.

* Corresponding author.

# equal contributions

Supporting Figures

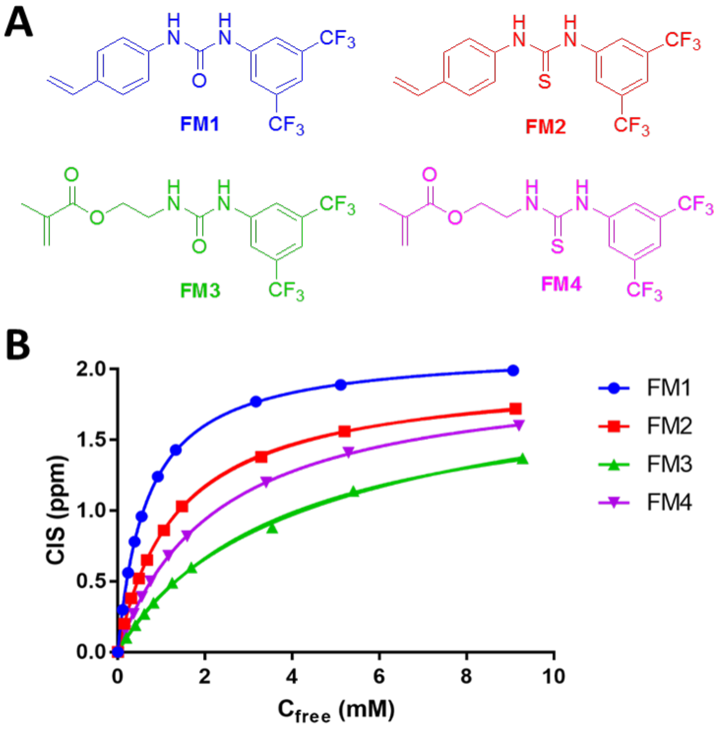

Figure S1. (A) Structures of functional monomers FM1-FM4 and (B) Plot of complexation induced shift (*CIS*) versus free concentration of guest (C_{free}) fitted to 1:1 interaction model for ^{1}H MNR titrations of FM1-FM4 with tetrabutylammonium hydrogen-1-naphtyl phosphate (TBAHNP) in DMSO-d_6 at 25 °C.

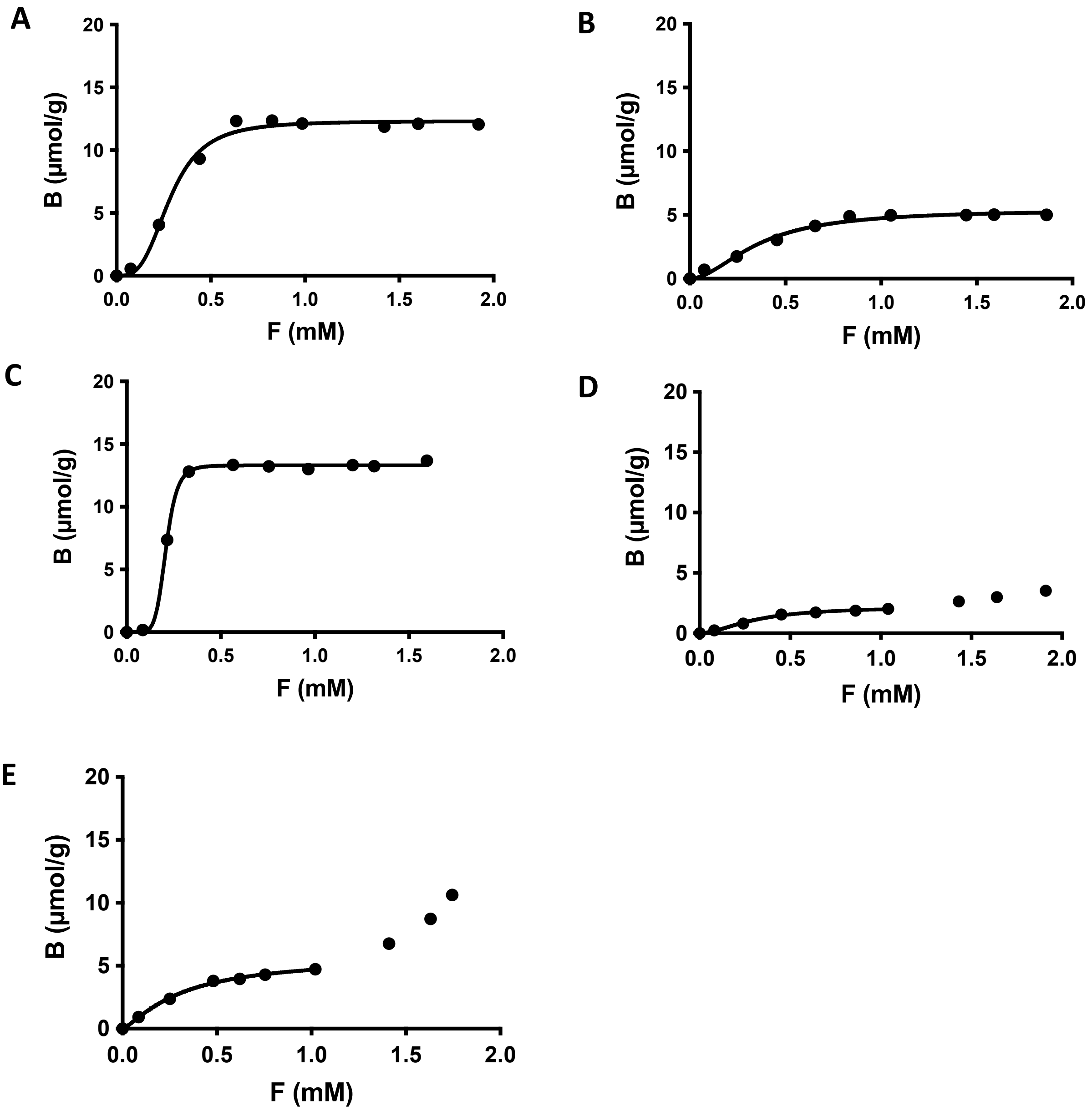

Figure S2. Corrected equilibrium binding isotherms for PPA·2PMP binding to polymer P1 (A), P9 (B), P10 (C), P11 (D) and P12 (E) in MeCN.

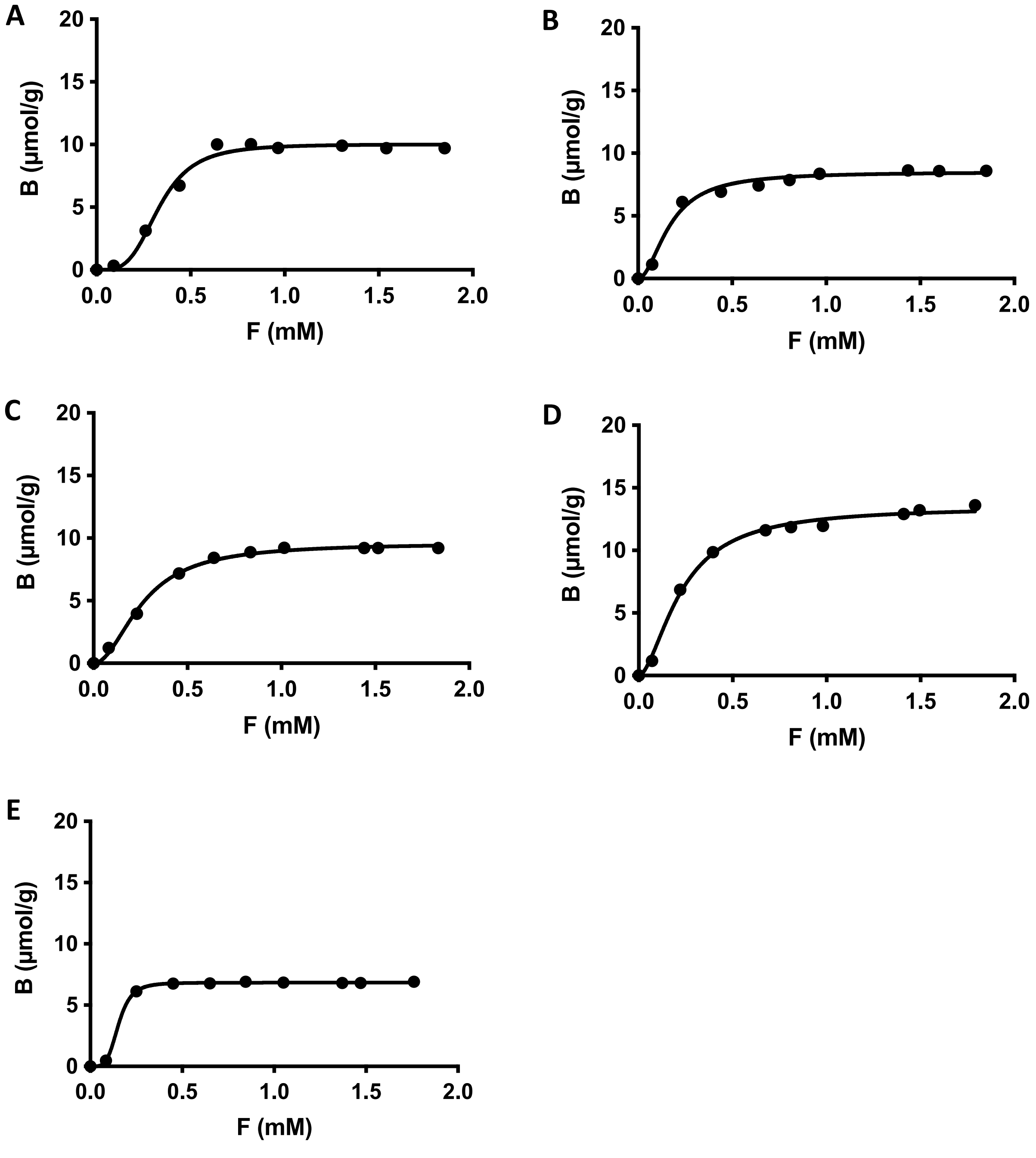

Figure S3. Corrected equilibrium binding isotherms for PPA·2Na binding to polymer P1 (A), P9 (B), P10 (C), P11 (D) and P12 (E) in 1M NaCl.

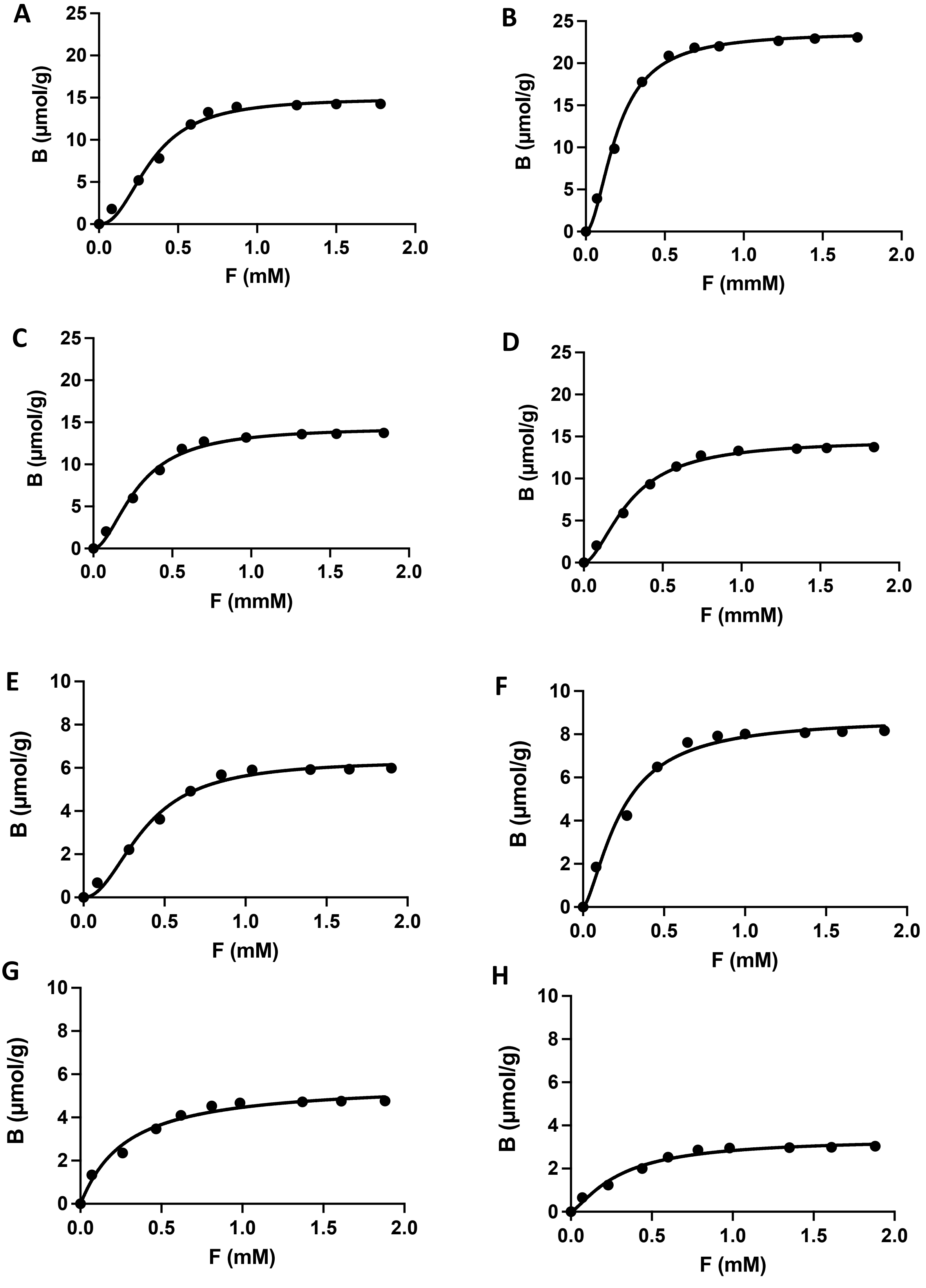

Figure S4. Corrected equilibrium binding isotherms for PPA·2Na (A-D) and PSA·Na (E-H) binding to polymer P11 (A,B,E,F) and P12 (C,D,G,H) in buffer pH 9 in absence (A,C,E,G) and presence (B,D,F,H) of 1M NaCl.

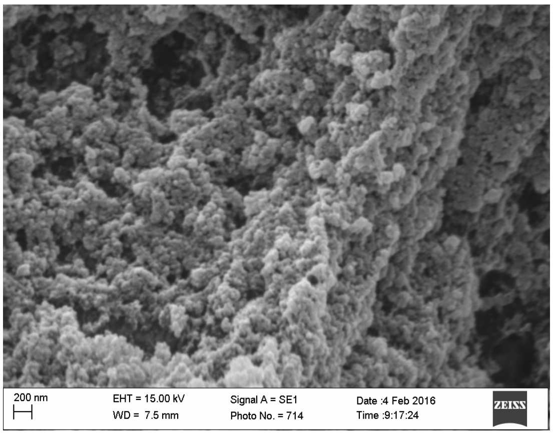
200 nm
EHT = 15.00 kV
WD = 7.5 mm
Signal A = SE1
Photo No. = 714
Date :4 Feb 2016
Time :9:17:24
ZEISS

A

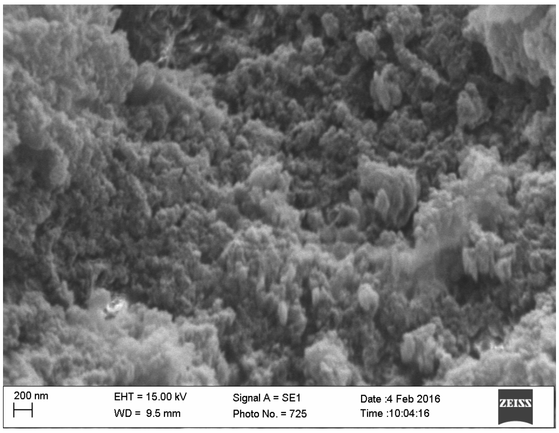
200 nm
EHT = 15.00 kV
WD = 9.5 mm
Signal A = SE1
Photo No. = 725
Date :4 Feb 2016
Time :10:04:16
ZEISS

B

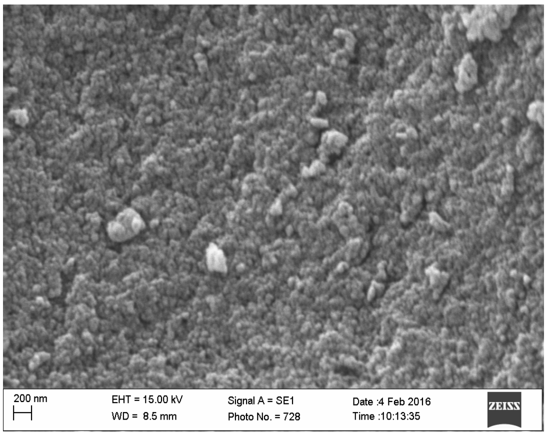
200 nm
EHT = 15.00 kV
WD = 8.5 mm
Signal A = SE1
Photo No. = 728
Date :4 Feb 2016
Time :10:13:35
ZEISS

C

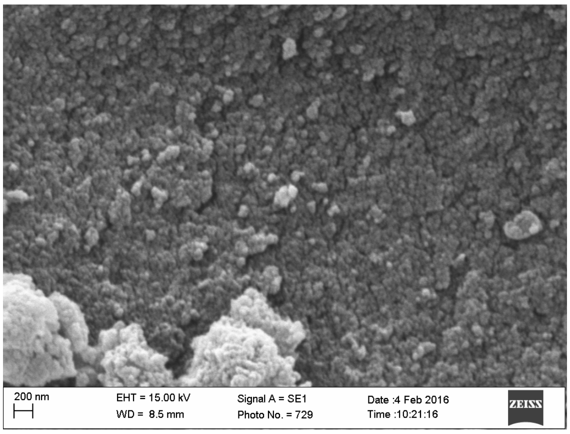
200 nm
EHT = 15.00 kV
WD = 8.5 mm
Signal A = SE1
Photo No. = 729
Date :4 Feb 2016
Time :10:21:16
ZEISS

D

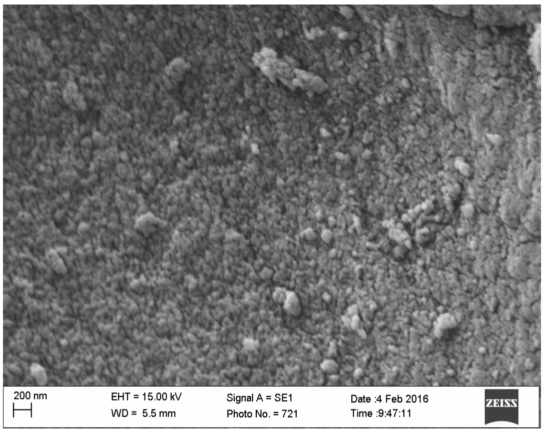
200 nm
EHT = 15.00 kV
WD = 5.5 mm
Signal A = SE1
Photo No. = 721
Date :4 Feb 2016
Time :9:47:11
ZEISS

E

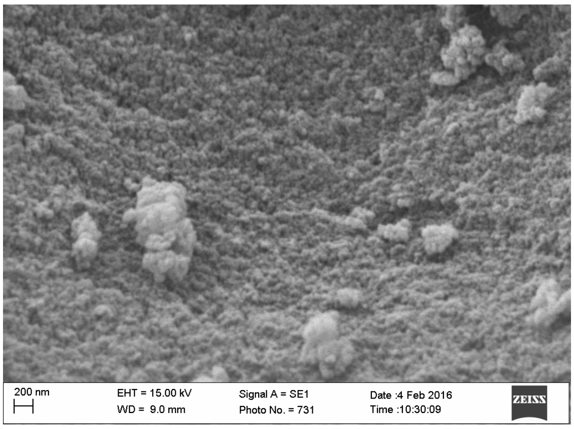
200 nm
EHT = 15.00 kV
WD = 9.0 mm
Signal A = SE1
Photo No. = 731
Date :4 Feb 2016
Time :10:30:09
ZEISS

F

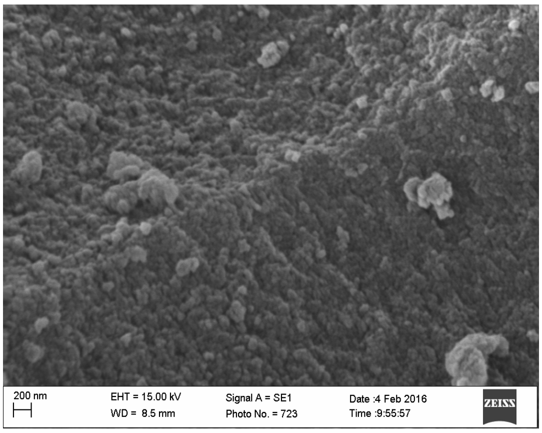
200 nm
EHT = 15.00 kV
WD = 8.5 mm
Signal A = SE1
Photo No. = 723
Date :4 Feb 2016
Time :9:55:57
ZEISS

G

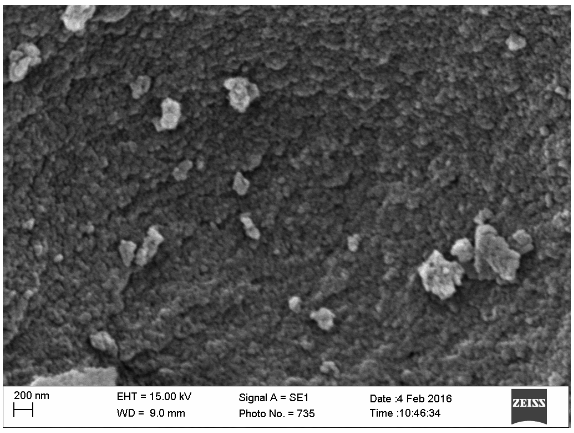
200 nm
EHT = 15.00 kV
WD = 9.0 mm
Signal A = SE1
Photo No. = 735
Date :4 Feb 2016
Time :10:46:34
ZEISS

H

Figure S5. Representative optical microscopic images of 25-36 μm particle size fractions of a) P9, b) P_N9, c) P11, d) P_N11, e) P12, f) P_N12, g) P13, h) P_N13. Scale bar indicates 200 nm.

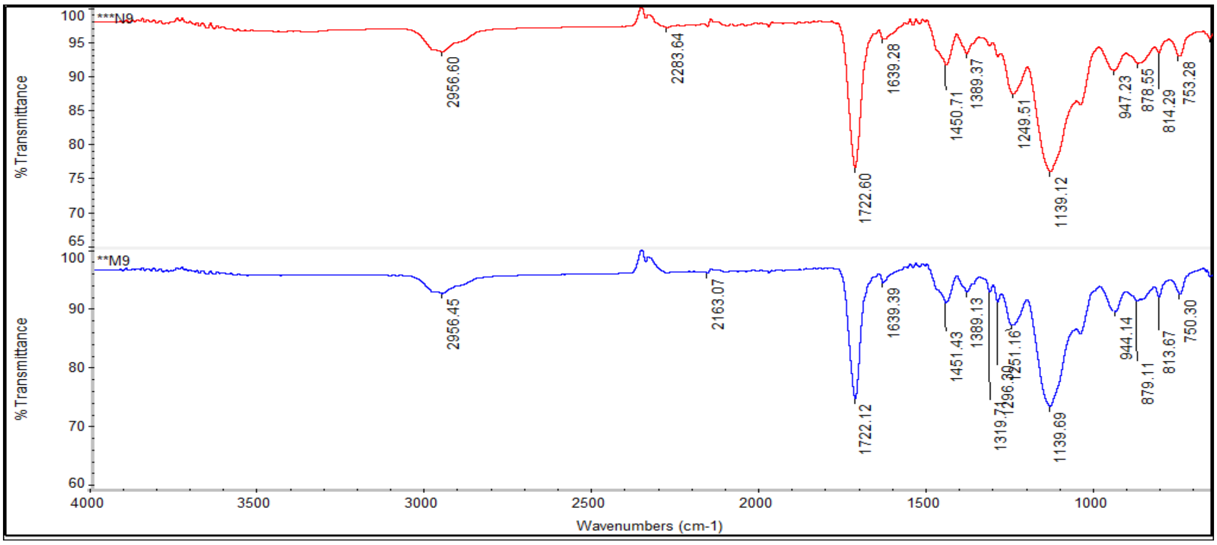

A.

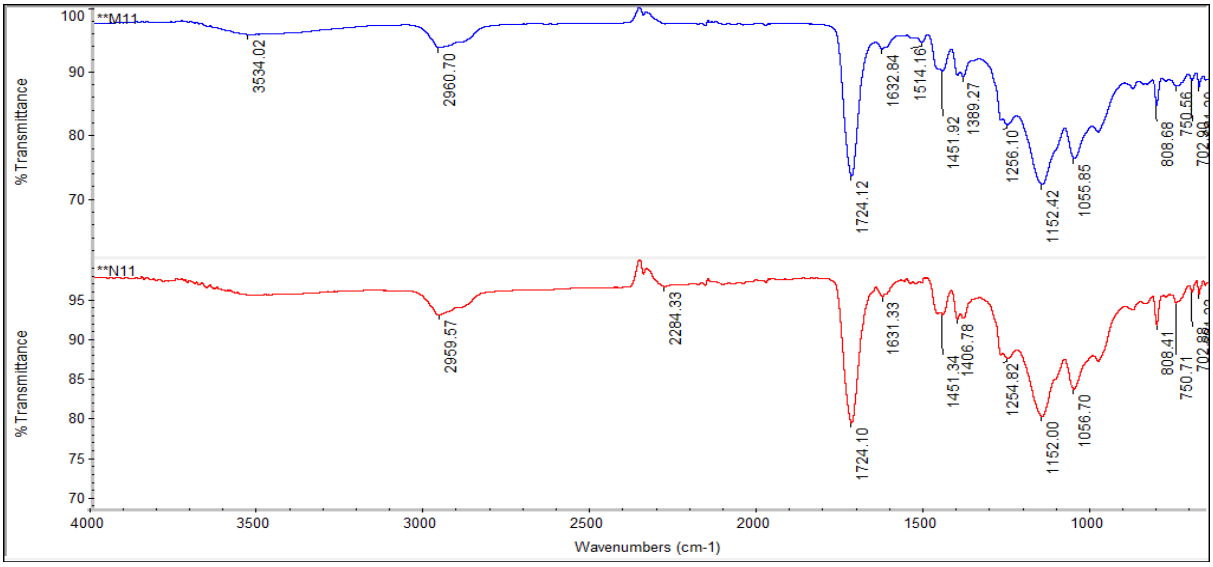

B.

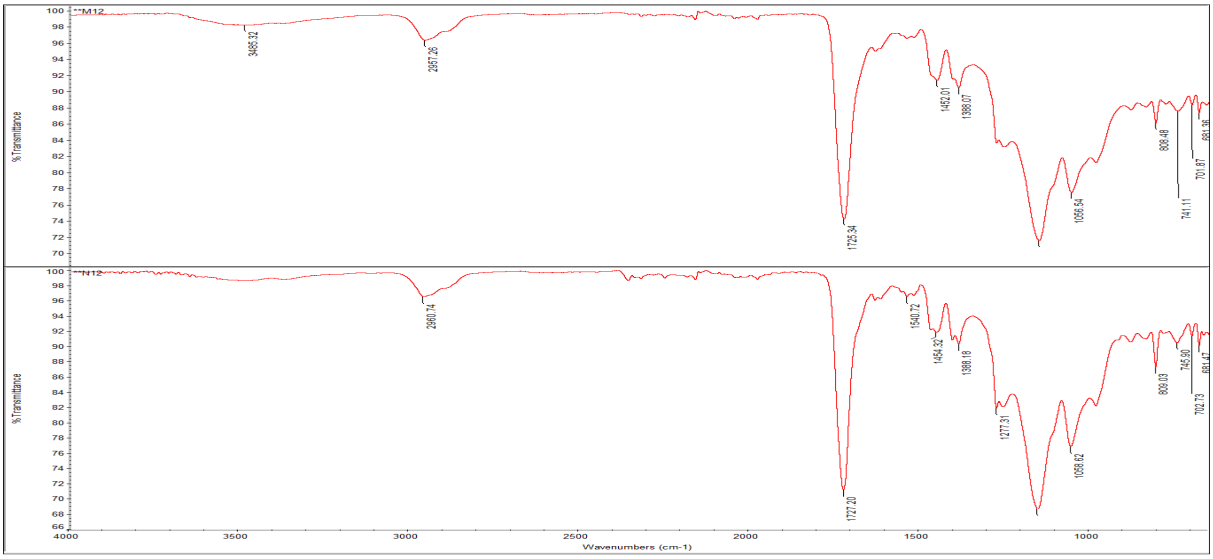

C.

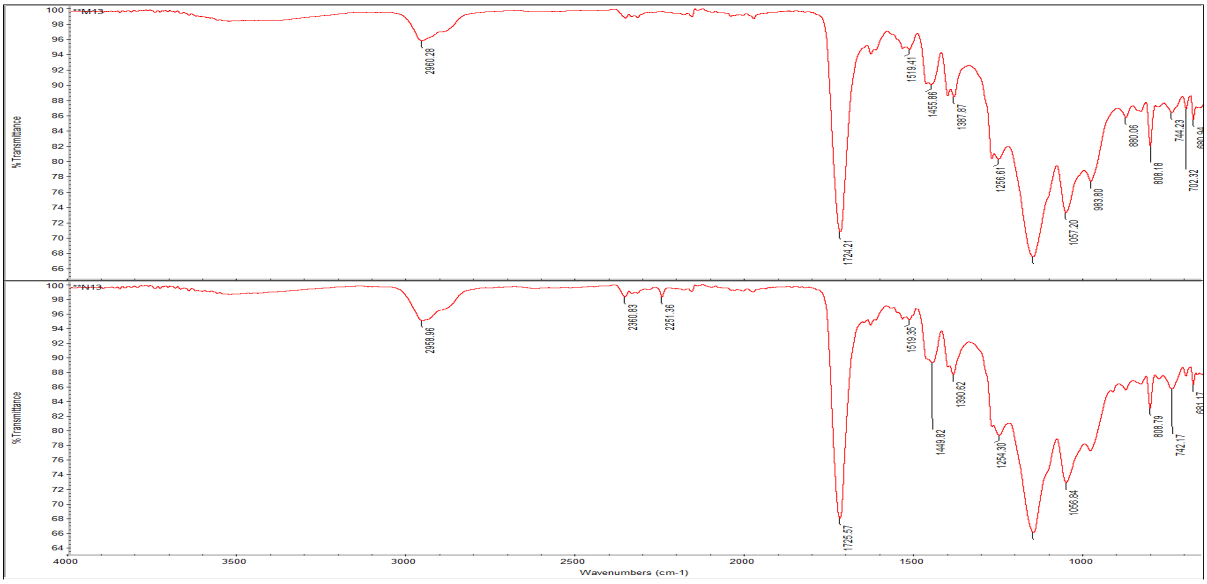

D.

Figure S6. Transmission infrared spectra (KBr) of MIPs (upper spectra) and NIPs (lower spectra) of (A) P9/PN9, (B) P11/PN11, (C) P12/PN12, (D) (P13/PN13. The spectra show the following characteristic bands: 3476 cm^{-1}, 3402 cm^{-1}: free CO-NH group and N-H stretching respectively, 3000 cm^{-1}: C-H stretch, 1700 cm^{-1}: C=O stretch.

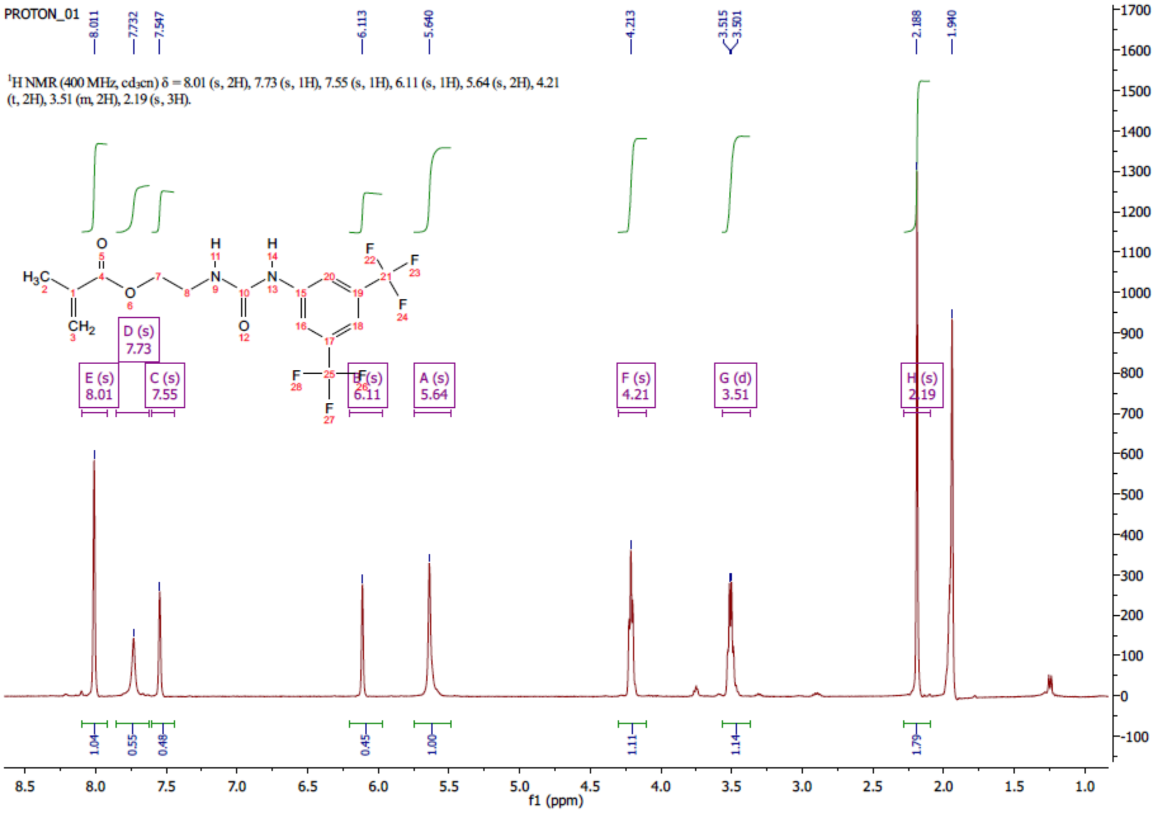

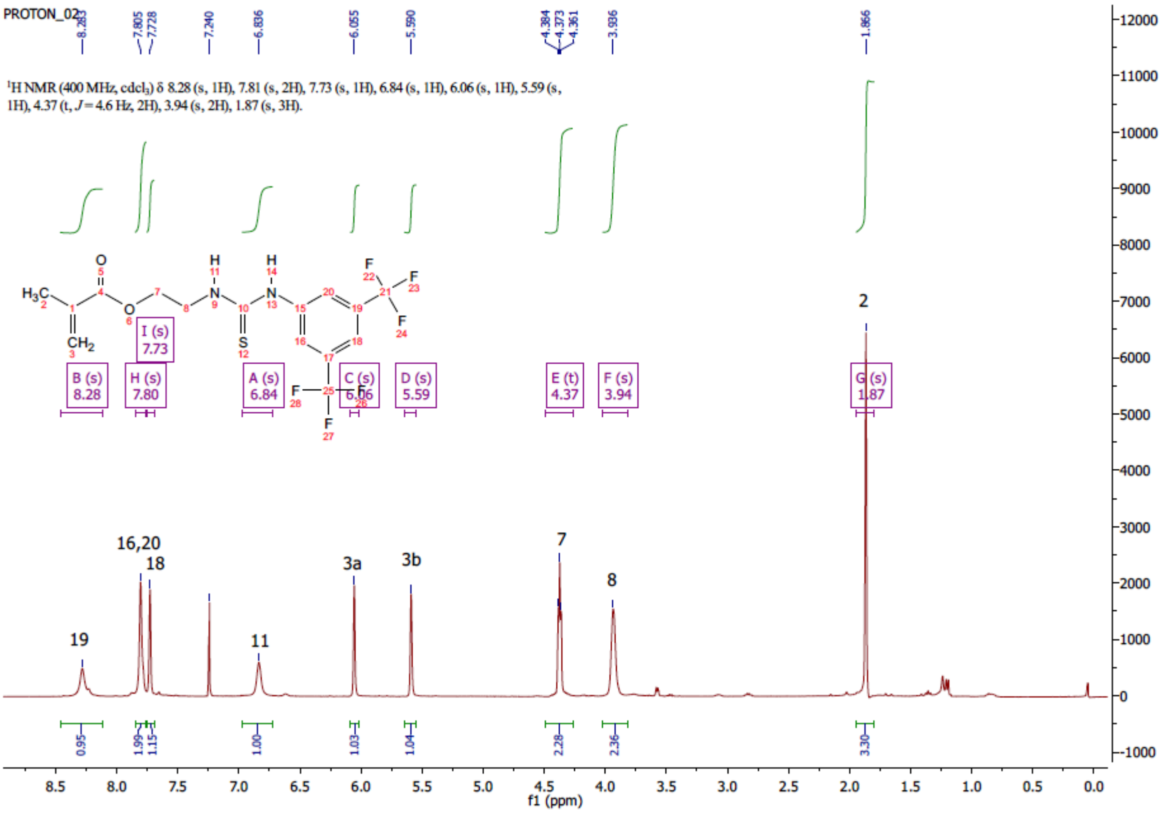

Figure. S7. ^{1}H NMR spectra (400 MHz, $CDCl_3$) of urea and thiourea monomers 3 (top) and 4 (bottom).

Supporting Tables

Table S1. Association constants (K_a) and binding capacities (B_{max}) of PPA·PMP on PPA imprinted polymers in MeCN

Polymer	Anion	K_a (x 10^3 M^{-1})	B_{max} (µmol/g)	R^2
P18	BA	2.2 ± 0.4	29 ± 1.7	0.974
P_N18	BA	1.7 ± 0.5	18 ± 1.9	0.934
P21	PPA	4.3 ± 0.4	65 ± 1.5	0.995
PN21	PPA	4.5 ± 2.9	17 ± 2.5	0.683

Table S2. Association constants (K_a) and binding capacities (B_{max}) of PPA·PMP on PPA imprinted polymers in MeCN

Polymer	Anion	K_a (x 10^3 M^{-1})	B_{max} (µmol/g)	R^2	h
P1	PPA	3.5 ± 0.2	12 ± 0.3	0.993	3.2 ± 0.4
P9	PPA	2.8 ± 0.3	5.4 ± 0.3	0.986	1.9 ± 0.3
P10	PPA	4.8 ± 0.1	13 ± 0.1	0.999	7±1
P11	PPA	3.3 ± 0.3	2.2 ± 0.1	0.996	1.8 ± 0.3
P12	PPA	3.2 ± 0.4	5.7 ± 0.4	0.998	1.3 ± 0.1

Table S3. Association constants (K_a) and binding capacities (B_{max}) of PPA·PMP and PSA·PMP on PPA imprinted polymers in 1M NaCl in water.

Polymer	Anion	K_a (x 10^3 M^{-1})	B_{max} (µmol/g)	R^2	h
P1	PPA	3.0±0.1	10±0.2	0.990	3.6±0.6
P9	PPA	5.9±0.5	8.5±0.2	0.989	1.9±0.3
P10	PPA	3.8±0.2	9.6±0.2	0.997	2.0±0.2
P11	PPA	4.4±0.3	13±0.3	0.996	1.7±0.2
P12	PPA	6.5±0.1	6.8±0.02	0.999	4.4±0.1
P11	PSA	4.7±0.2	7.4±0.12	0.997	1.9±0.2

Table S4. Properties of artificial urine spiked with glyphosate before and after solid phase extraction using cartridges packed with P11, P12 and P13[a]

Parameter	Unit	Contaminate urine	Polymers					
			P11	PN11	P12	PN12	P13	PN13
**pH**	……..	6.25 ± 0.10	7.8 ± 0.40	6.9 ± 0.20	7.4 ± 0.30	7.0 ± 0.25	7.2 ± 0.37	7.6 ± 0.41
**Color**	……...	Dark yellow	Pale yellow	Pale yellow	Pale yellow	Pale yellow	Pale yellow	Pale yellow
**EC**	**mS**	9.78 ± 0.20	6.9 ± 0.10	8.2 ± 0.15	2.1 ± 0.06	8.0 ± 0.40	2.3 ± 0.08	7.4 ± 0.30
**TDS**	**g/l**	6.0 ± 0.50	4.2 ± 0.24	5.0 ± 0.32	1.3 ± 0.04	4.8 ± 0.21	1.4 ± 0.07	4.0 ± 0.22
**COD**	**mg O_2 /L^{-1}**	17040 ± 60	9056 ± 20	9961 ± 50	3018 ± 10	9900 ± 40	3200 ± 15	10160 ± 55
**Glyphosate**	**mM**	0.6 ± 0.05	0.42 ± 0.02	0.51 ± 0.03	0.2 ± 0.01	0.5 ± 0.04	0.23 ± 0.02	0.48 ± 0.04

a) Average values from 3 replicate experiments.
